# Friendships and Coping Among Adolescents with LGBTQ+ Parents

**DOI:** 10.3390/bs16060977

**Published:** 2026-06-12

**Authors:** Jacob S. Withrow, Nita U. Kulkarni, Rachel H. Farr

**Affiliations:** Department of Psychology, University of Kentucky, Lexington, KY 40508, USA; nita.kulkarni@uky.edu (N.U.K.); rachel.farr@uky.edu (R.H.F.)

**Keywords:** adolescence, LGBTQ+ parents, coping, mental health, peer relationships

## Abstract

Adolescents with LGBTQ+ parents and LGBTQ+ adolescents navigate unique social and identity-related challenges as compared to those without minoritized sexual and/or gender identities. Adolescents with LGBTQ+ parents (regardless of their own sexual or gender identity) and adolescents who personally identify as LGBTQ+ are distinct populations, though they sometimes overlap. Research on adolescents with LGBTQ+ parents has often focused on parent–adolescent relationships and family structures. How do friends help youth cope with identity-based minority stressors, like peer microaggressions, bullying, and exclusion, common for those with minoritized identities? Friendships are developmentally pivotal during adolescence, shaping social competence, identity exploration, and psychological adjustment. Grounded in ecological systems, social learning, and minority stress theories, we sought to understand how friendships relate to mental health and coping in adolescents with LGBTQ+ parents. This cross-sectional quantitative study included 98 adolescents (ages 12–19) with LGBTQ+ parents in the U.S., recruited via community sampling and Prolific. Higher-quality peer attachment, conceptualized by trust, communication, and alienation in close friendships, was associated with lower depression and greater social competence, but not associated with anxiety or adaptive coping (after accounting for avoidant coping). Avoidant coping was most strongly associated with poorer mental health. This study, with implications for practice, emphasizes the importance of peer relationships for adolescents with LGBTQ+ parents—particularly how high-quality friendships offer important possible protection via social competence and against depression—while also highlighting the complex interplay between friendships, coping, and adjustment.

## 1. Introduction

The demographic fabric of families in the United States (U.S.) has undergone notable changes over the past several decades. Contributing to this evolution is the increasing visibility, normalization, and legal recognition of families headed by lesbian, gay, bisexual, transgender, and queer (LGBTQ+) parents. For adolescents growing up in LGBTQ+ families, their development unfolds within a complex and often contradictory social context. Adolescents with LGBTQ+ parents and adolescents with LGBTQ+ identities navigate unique social and identity-related challenges, as compared to those with cisgender-heterosexual (cis-het) identities and/or who have parents with cis-het identities. While legal milestones like marriage equality have provided tangible protections, they have not eradicated societal stigma and cisgender-heteronormative bias that is often the dominant mindset in many areas. In fact, this societal stigma and experiences with discrimination can have negative effects on the mental health of adolescents amid an already tumultuous time (Farr et al., 2016; Rivas-Koehl et al., 2023). Adolescents within families headed by LGBTQ+ parents, therefore, often have to navigate a unique developmental pathway from childhood to young adulthood, shaped not only by the universal tasks of identity formation, autonomy development, and academic achievement, but also by experiences tied to their family structure. (Breshears, 2011; Farr et al., 2016). These experiences exist on a spectrum, ranging from neutral or mildly challenging experiences, such as curious questions from peers about their family, to overtly hostile interactions, including teasing, bullying, or social exclusion, especially if adolescents also hold (LGBTQ+) identities themselves (Kuvalanka & Goldberg, 2009; N. U. Kulkarni et al., 2026). Given this reality, these youth often develop a keen awareness of societal norms and differences from a young age to assist in their own development into maturity and adulthood. For example, one study found that adolescents with lesbian mothers exhibited significantly higher social, school, and overall competence, as well as lower rates of social problems and externalizing behaviors, compared to age-matched peers with cis-het parents from a U.S. sample (Gartrell & Bos, 2010). Such findings suggest that these adolescents may possess strengths that support positive development. However, the existing literature has disproportionately focused on family-level dynamics, leaving a critical gap in understanding how extra-familial relationships, particularly friendships, may contribute to these positive outcomes. The present study addresses this gap by examining the protective functions of peer relationships among adolescents with LGBTQ+ parents.

### 1.1. The Dominance of Family-Level Studies and the Need for a Peer Focus

Adolescence is universally recognized in developmental science as a period of profound biological, psychological, and social change (Casey et al., 2008; Nelson et al., 2016). While often characterized by exploration and growing competence, this developmental stage also constitutes a period of heightened vulnerability for the onset of mental health challenges. Rates of internalizing disorders, such as depression and anxiety, significantly increase during this time, linked to factors including hormonal fluctuations, neurodevelopment, and increasing social and academic pressures, which can be buffered by familial and parent relationships (Patton, 2016). Against this backdrop of universal risk, the family environment is consistently identified as one of the most powerful determinants of an adolescent’s psychological well-being (Hamza & Willoughby, 2011).

For adolescents with LGBTQ+ parents, understanding mental health requires acknowledging both typical developmental trajectories and the specific social context in which they develop. Decades of research have demonstrated that these children develop in just as healthy and adaptive ways as their peers from cisgender, heterosexual parent families (Biblarz & Stacey, 2010; Fedewa et al., 2014). A significant body of work has effectively focused on family-level dynamics, consistently finding that factors such as family processes, parental psychological well-being, and the quality of the parent–child relationship are robust predictors of positive child outcomes, often outweighing the importance of family structure itself (Frosch et al., 2019; Sumontha et al., 2017).

However, this focus on familial contexts, while being necessary and valuable to understanding these families, has inadvertently cast a shadow on other important developmental contexts. The research narrative has been dominated by how parental relationships and family functioning correlate with mental health characteristics, leaving a significant gap in understanding the extra-familial social worlds of these youth. As adolescents spend increasing time with peers and seek autonomy, their friendship networks become a primary arena for social learning, identity exploration, and emotional support (Brown & Larson, 2009). The relative absence of research into these peer relationships for youth with LGBTQ+ parents represents a gap in the literature that we seek to address. While research rightly supports that these adolescents show positive adjustment, it has often done so by focusing on the social stressors they face, such as discrimination or a lack of safe spaces, rather than investigating the potential positive buffers, like high-quality friendships, that are readily available in their developmental environment.

### 1.2. Key Theoretical Frameworks: Grounding in Developmental Science

To understand the potential role of peer relationships for adolescents with LGBTQ+ parents, it is essential to ground this area of study within established developmental theories. These frameworks provide the scaffolding for hypothesizing how different systems interact to influence adolescent outcomes and allow for a more nuanced examination of our key constructs.

Ecological Systems Theory (Bronfenbrenner & Morris, 2007) posits that development is shaped by a series of nested environmental systems, from the immediate microsystem (e.g., family, school, peers) to the broader macrosystem (e.g., cultural norms, laws). This theory underscores that adolescents with LGBTQ+ parents develop within a unique ecological niche. Their experiences are not only influenced by direct interactions with parents and friends (microsystem) but also by the connections between these systems (mesosystem), such as how parents manage their child’s peer relationships (Goldberg & Smith, 2024). For adolescents, the peer microsystem becomes increasingly central, and supportive friendships represent a key developmental asset that may directly influence well-being (Bronfenbrenner & Morris, 2007; Erikson, 1980). Furthermore, societal attitudes toward LGBTQ+ people (macrosystem) create a context of either support or stigma that permeates all other systems (Ogolsky et al., 2019; Patterson, 2017). This theory forces us to look beyond the family unit to see the adolescent as embedded in a wider social world that contributes to their overall adaptation.

Minority Stress Theory (Brooks, 1981; Meyer, 1995), originally developed for sexual minority communities, provides a vital lens for understanding the unique social challenges adolescents with LGBTQ+ parents may face. The theory posits that health disparities are driven by distal stressors (e.g., experiences of discrimination, victimization) and proximal stressors (e.g., internalized stigma, expectation of rejection). Specifically, minority stress has been linked to increased depression and anxiety symptoms among LGBTQ+ parents (Siegel et al., 2025). Adolescents in LGBTQ+-parented families may experience minority stress related to their family identity, subsequently leading to increased depression and anxiety, regardless of their own sexual orientation or gender identity (Sumontha et al., 2017; Farr et al., 2016). Peer support has been found to be correlated with decreased minority stress among LGBTQ+ individuals; in fact, higher peer support was linked with lower general stress and higher resilience (Haas & Lannutti, 2019). Thus, social support and high-quality friendships could potentially mitigate the psychological toll or minority stress among adolescents with LGBTQ+ parents (Haas & Lannutti, 2019). Social Learning Theory (Bandura & Walters, 1977) offers a mechanism for how coping strategies might be developed and reinforced. Adolescents learn not only from direct instruction but also by observing and modeling the behaviors of others. Within the family, they may learn specific coping skills from their parents, who have often navigated their own minority stressors (Goldberg & Smith, 2024). Crucially, this learning extends to the peer group, where adolescents can observe and adopt their friends’ strategies for managing social challenges, providing an arsenal of potential coping mechanisms. Friends serve as important models; observing a friend navigate a challenging social situation can provide a repertoire of adaptive coping responses. When adolescents from LGBTQ+ parent families experience discrimination due to their family identity, this social modeling could be especially helpful in demonstrating adaptive coping strategies. Further, social competence, or the ability to effectively evaluate a social situation and react appropriately, can be honed through supportive peer relationships (Bandura & Walters, 1977). Social competence has been linked with better adolescent adjustment (Dennis et al., 2007). Therefore, from social learning, one can predict that adolescents who have strong peer relationships may also have strong coping skills (as modeled by peers), higher social competence, and overall positive adjustment (Bandura & Walters, 1977; Dennis et al., 2007). The core constructs of this study are directly related to these theoretical foundations. Societal stigma and minority stress can negatively impact the well-being of adolescents in LGBTQ+ families, and social support, particularly from peers, is theorized to be a critical protective factor that can directly influence well-being and buffer stress.

### 1.3. An Essential Developmental Context for Youth with LGBTQ+ Parents

While the importance of parental support is well-documented and forms the bedrock of much developmental research, the adolescent stage is fundamentally defined by a developmental push beyond primary parental connections. This period marks a strategic shift in attachment and support-seeking behaviors, where peers become a central source of intimacy, companionship, and validation (Allen et al., 2020). This is not to diminish the role of parents, but to recognize that the social ecosystem expands dramatically. For any adolescent, high-quality friendships are robustly associated with a multitude of positive outcomes, including better psychological adjustment, higher self-esteem, greater social competence, and lower rates of anxiety and depression (Bagwell & Schmidt, 2011; Bukowski et al., 2011). Friends provide a critical “practice field” for navigating complex social situations, experimenting with identity, and developing autonomy. They also offer a safe haven for disclosing fears, insecurities, and experiences that adolescents may be reluctant to share with their parents, for fear of judgment or causing concern (Nickerson & Nagle, 2005).

For adolescents with LGBTQ+ parents, these friendships may hold unique, amplified significance, functioning as a potentially powerful protective system. Research on queer parent family socialization has found positive associations with adolescent social competence and adaptive coping, suggesting that family-level discussions about identity and discrimination equip youth with skills that likely extend into their peer relationships (N. Kulkarni & Farr, 2026). Furthermore, longitudinal research indicates that adolescents in lesbian-parent families often demonstrate strong psychological adjustment, with family characteristics helping to counteract the negative effects of stigmatization (Gartrell & Bos, 2010). They can serve as a vital buffer against external stressors, offering normative social validation and a crucial respite from potential stigma or microaggressions related to their family structure (Obeldobel & Kerns, 2021). A supportive friend who accepts their family without question can directly counteract and neutralize negative experiences of social exclusion or othering. The simple act of having a friend over to the house of a two-mother or two-father family, where the family dynamic is treated as completely normal, can be a profoundly affirming experience that builds resilience. Furthermore, for the subset of these adolescents with LGBTQ+ parents who also hold a sexual or gender diverse (SGD) identity themselves—or “second-generation” queer youth (Kuvalanka & Goldberg, 2009)—these friendships can be a doubly crucial source of identity affirmation and a gateway to a broader LGBTQ+ community, which is itself linked to greater resilience and positive identity development (Craig et al., 2015; Fish & Russell, 2022). Indeed, prior research has specifically examined peer relationships among adolescents with same-sex parents, finding that those whose parents reported closer relationships with them had higher quality peer relations, more friends in school, and greater centrality within their friendship networks (Wainright & Patterson, 2008). This work demonstrates that family processes—rather than family structure—are associated with positive peer outcomes, highlighting the importance of examining how parent–child relationships may scaffold the development of supportive friendships (Wainright et al., 2004). In these cases, peers may provide not only general emotional support but also specific, identity-affirming support that is uniquely tailored to the challenges of navigating multiple minoritized statuses.

### 1.4. Adolescent Friendships Are Vital

Friendships, adjustment, and coping among adolescents in families with LGBTQ+ parents are shaped through a complex interaction of socialization and communication. Building upon the established context of this growing population, the theoretical frameworks of ecological systems, social learning, and minority stress, and the identified gap concerning extra-familial peer relationships, this project sought to provide a focused empirical investigation. The primary objective was to quantitatively examine how the self-described quality and nature of friendships contribute to the mental health and resilience of adolescents with LGBTQ+ parents. Specifically, we sought to test whether high-quality peer attachment would serve a protective (buffering) function, weakening the association between minority stress-related coping (i.e., avoidant coping) and psychological distress as measured through constructs of depression and anxiety. We hypothesized that adolescents reporting stronger peer attachment would exhibit lower levels of depression and anxiety and that friendship quality would moderate the relationship between avoidant coping and mental health outcomes. It was further anticipated that friendship quality would serve as a significant moderating factor, buffering the negative impact of potential minority stress on mental health outcomes. By moving beyond the family-centric focus, this research aims to illuminate a potentially important, yet under-analyzed, developmental asset in these adolescents’ lives.

To address this gap, a secondary aim was pursued. We aimed to highlight how the protective role of peer support may intersect with other key identities, such as age, race, gender, and sexual identities, acknowledging that adolescents with one or more minoritized identities may experience and leverage social support in distinct ways (Graham et al., 2014; Bos & Sandfort, 2015). We also sought to contribute to the refinement of developmental theories by testing their applicability to the specific ecological niche of youth in LGBTQ+-parented families, particularly concerning the mesosystemic links between family and peer contexts.

## 2. Materials and Methods

### 2.1. Research Design and Data Source

This study employed a quantitative, correlational research design to analyze existing data from the Queer Parents Adolescent Lives (QPAL) SurveyThe original QPAL study was a national, longitudinal survey designed to examine the family dynamics, social experiences, and well-being of LGBTQ+ parents and their adolescent children. For the current project, quantitative data from adolescents with LGBTQ+ parents were analyzed.

### 2.2. Participants

The sample consisted of 98 adolescents (ages 12–19 years; M = 15.7 years, SD = 2.1) who had at least one parent identifying as LGBTQ+ and lived in the U.S. For the purposes of this study, ‘parent’ was defined broadly to include biological, adoptive, and step-parents whom the adolescent identified as primary caregivers. Inclusion criteria for the study generally reflected that adolescents had resided with an LGBTQ+ parent for a minimum of one year while growing up. They also represented different U.S. regions (41% South, 28% West, 16% Northeast, and 14% Midwest). Participants were recruited nationally through LGBTQ+ family organizations, online communities, and Prolific, an online research platform (www.prolific.com). Regarding the adolescents’ own identities, 43% identified as cisgender women, 40% as cisgender men, and 17% as transgender, nonbinary, or gender-diverse. In terms of sexual orientation, 62% identified as heterosexual, 25% as bisexual, 8% as lesbian or gay, and 5% used other labels (e.g., pansexual, queer). The racial/ethnic composition of the adolescent sample was 60% people of color (POC) and 40% white. Demographic information was collected via questions within the QPAL survey. For race/ethnicity, participants were instructed to state in a free-response form how they identify. These included White, Black or African American, Hispanic/Latino, Asian or Pacific Islander, Native American, Middle Eastern, and an option to write in another identity. Participants who stated multiple categories were coded as Multiracial. For gender identity, participants were also allowed to write exactly how they identified, which included cisgender man, cisgender woman, transgender man, transgender woman, nonbinary, genderfluid, and an open-ended option; responses were later collapsed into cisgender and gender-expansive categories for analysis. Sexual orientation was assessed with a free-response measure, allowing participants to list a sexual orientation they felt was most salient (e.g., heterosexual, gay, lesbian, bisexual, pansexual, queer, asexual). Missing data were permitted; no forced responses were implemented. Cases with missing data were handled using listwise deletion in analyses.

Refer to Table 1 for additional demographic details.

### 2.3. Procedure

After receiving IRB approval for QPAL, advertising, recruitment, and data collection for the study began. By clicking a link found on online advertisements, scanning a QR code on fliers, or directly connecting via Prolific, those interested in participating in the study gained access to an eligibility survey via Qualtrics. Once a member of the research team screened the participant’s eligibility, eligible participants were sent a link to the main Qualtrics survey. After completing this survey, participants were sent a $35 Amazon e-gift card and were debriefed on the study via email.

### 2.4. Measures

#### 2.4.1. Inventory of Parent and Peer Attachment (IPPA)—Peer Attachment Subscale

The quality of the adolescent’s close friendships was assessed using the peer attachment subscale of the Inventory of Parent and Peer Attachment (Armsden & Greenberg, 1987). This widely used 25-item self-report measure evaluates a parent’s and adolescent’s perceived trust in, communication with, and degree of alienation from their primary peer relationships. This project solely focused on peer attachment within our adolescent sample. Participants responded to statements such as “My friends understand me” and “I feel my friends are good friends” using a 5-point Likert scale ranging from 1 (Almost never or never true) to 5 (Almost always or always true). After reverse-scoring the 8 items from the alienation subscale, all item scores were summed to create a total peer attachment score. Higher scores on this scale reflect greater perceived security, trust, and positive communication within peer relationships, indicating higher-quality friendships. The IPPA peer attachment subscale has demonstrated strong reliability and validity in adolescent populations. In the current sample, the scale demonstrated a strong internal consistency (α = 0.89).

#### 2.4.2. Center for Epidemiological Studies Depression Scale (CES-D)

Symptoms of depression were measured using the 20-item Center for Epidemiological Studies Depression Scale (CES-D; Radloff, 1977). This scale is a widely used self-report instrument designed to assess the frequency of depressive symptoms in the general population over the past week. It captures core dimensions of depressive affect, including feelings of sadness, hopelessness, and loneliness, as well as somatic complaints, interpersonal difficulties, and positive affect (reverse-scored). Participants responded to items such as “I was bothered by things that usually don’t bother me” and “I felt depressed” using a 4-point Likert scale ranging from 0 (Rarely or none of the time [less than 1 day]) to 3 (Most or all of the time [5–7 days]). After reverse-scoring the four positively worded items, all item ratings were summed to create a total depression score, with scores ranging from 0 to 60. Higher scores indicate greater severity of depressive symptomatology. The CES-D has established strong reliability and validity across diverse populations, and therefore, this translates to measuring distress within this project’s sample of adolescents. Within the current study, the scale demonstrated acceptable reliability (*a* = 0.78).

#### 2.4.3. Generalized Anxiety Disorder Scale (GAD)

Symptoms of generalized anxiety were assessed using the 7-item Generalized Anxiety Disorder scale (GAD-7; Spitzer et al., 2006). This brief self-report measure asks participants how often they have been bothered by common anxiety symptoms over the past two weeks. Items include “Feeling nervous, anxious, or on edge” and “Not being able to stop or control worrying.” Responses were recorded on a 4-point Likert scale from 0 (Not at all) to 3 (Nearly every day). The seven-item scores were summed to produce a total anxiety score, where higher scores represent greater anxiety severity. The GAD-7 is a reliable and valid measure for screening anxiety in adolescent and adult populations, which serves as another piece of distress measurement for our adolescent sample. The reliability of the GAD within our sample was shown to be strong (*a* = 0.92).

#### 2.4.4. Youth Self-Report (YSR)—Social Competence Scales

To assess social competence, we used select scales from the Youth Self-Report (YSR; Achenbach & Rescorla, 2001; Song et al., 1994), a well-validated, norm-referenced instrument for youth aged 11–18. For this study, we focused on the social competence scale. The social competence scale is a 6-item subscale assessing the adolescent’s involvement in activities (e.g., sports and jobs), social engagement (e.g., number of friends and quality of relationships), and overall relationships with themself and others (e.g., being able to get along with other children and do things alone). Each item was rated on a 0–2 or 0–3 rating scale, with higher numbers indicating more involvement. The values were then averaged to create a mean score, for which an age- and gender-normed T-score can be generated, where higher T scores are indicative of superior social competence and prosocial functioning. In the current sample, this scale demonstrated nearly acceptable internal consistency (α = 0.68). Since this reliability score is lower than the typically accepted value, our team conducted additional analyses, discussed in the limitations section below. All YSR scales have extensive evidence of reliability and validity, and the chosen areas within the YSR are pivotal to analyzing these adolescents’ social and adaptive functioning.

#### 2.4.5. Brief COPE (B-COPE) Inventory—Adaptive and Avoidant Coping Composites

Strategies for coping with stress were assessed using selected subscales from the Brief COPE Inventory (BCOPE; Carver, 1997), a 28-item multidimensional measure of coping responses. All items were rated on a 4-point scale from 1 (I haven’t been doing this at all) to 4 (I’ve been doing this a lot). Higher scores on each composite indicate a greater reported use of that coping style. For the purposes of this study, two composite categories were analyzed that represent overarching coping styles. The Adaptive Coping composite summed items from the active coping, planning, positive reframing, acceptance, and seeking emotional support subscales (14 items total). An example item is, “I’ve been trying to see it in a different light, to make it seem more positive.” In the current sample, this composite demonstrated good internal consistency (α = 0.88). The Avoidant Coping composite was formed by summing items from the denial, behavioral disengagement, and self-blame subscales (9 items total). An example item is, “I’ve been refusing to believe that it has happened.” In the current sample, this avoidant composite score demonstrated acceptable internal consistency (α = 0.73). The Brief COPE has been validated for use with adolescent samples (Fernández-Martín et al., 2022; Marakshina et al., 2025).

### 2.5. Analyses

#### 2.5.1. Data Preparation and Preliminary Analysis

All analyses were conducted using IBM SPSS Statistics (v. 31.0; IBM Corp., 2025). Prior to hypothesis testing, data were prepared by screening for missing values, which were addressed using listwise deletion for any incomplete cases. Descriptive statistics (means, standard deviations, and ranges) were computed for all primary study variables, including the predictor variable (peer attachment), the mental health outcome variables (depression and anxiety), the coping variables (adaptive and avoidant coping), the adjustment variable (social competence), and key demographic covariates (age, sexual identity, gender identity, and race).

#### 2.5.2. Bivariate Correlations

A Pearson correlation matrix was generated to examine initial associations among all primary continuous variables (i.e., peer attachment, depression, anxiety, social competence, adaptive coping, and avoidant coping).

#### 2.5.3. Regression Models

To further explore the nuances of peer attachment with regard to depression and anxiety and to test whether peer attachment is uniquely associated with outcomes after accounting for other relevant factors, three separate multiple linear regression analyses were conducted. Covariates showing significant bivariate correlations with the outcome variables were included in the respective models.

- Model 1: Predicting Depression. This model tested whether peer attachment statistically predicted lower levels of depression when accounting for age and avoidant coping.
- Model 2: Predicting General Anxiety. This model tested whether peer attachment predicted lower anxiety symptoms when accounting for age and avoidant coping.
- Model 3: Predicting Adaptive Coping. This model tested whether peer attachment predicted greater use of adaptive strategies beyond the influence of age and general social competence.

#### 2.5.4. Moderated Regression Analysis: Testing the Primary Buffering Hypothesis

To directly test the protective, buffering role of peer attachment between avoidant coping and depression, as derived from Minority Stress Theory (Brooks, 1981; Meyer, 1995), a hierarchical moderated regression was performed. With depression as the outcome variable, peer attachment was entered in the first step of hierarchical analysis along with age and avoidant coping as covariates. The second step of this hierarchical model had the interaction term between avoidant coping and peer attachment.

#### 2.5.5. Power Considerations

Although the sample size (*N* = 98) was sufficient for detecting moderate bivariate associations and main effects in regression models, it offered limited statistical power for detecting smaller effects, particularly interaction terms and subgroup differences. Given the modest cell sizes within demographic groups (e.g., gender-expansive youth, smaller racial categories), tests of moderation and identity-based comparisons were expected to be underpowered. As such, all moderation and subgroup analyses were interpreted cautiously, with emphasis on effect sizes rather than significance alone.

## 3. Results

### 3.1. Primary Analyses: Descriptives and Data Screening

Descriptive statistics were calculated for all primary study variables. Data for key variables were largely complete with little missingness: listwise Ns for analyses ranged from 87 to 98. Peer attachment showed good variability (*M* = 91.26, *SD* = 19.96) with scores falling in the moderate-to-high range relative to scale ranges, indicating that adolescents in this sample generally perceived their friendships as relatively trusting and supportive. Scores on the depression (*M* = 20.26, *SD* = 11.78) exceeded the established clinical cutoff of 16 or greater on the CES-D, suggesting that, on average, participants reported clinically significant levels of depressive symptomatology. Depression scores also displayed high variability. Anxiety (*M* = 7.04, *SD* = 5.64) indicates mild anxiety symptoms on average, though with considerable variability. Social competence scores (*M* = 41.57, *SD* = 12.03) indicated moderate levels in participants’ self-reported social engagement, involvement in activities, and adaptive functioning relative to age- and gender-normed expectations. For planned group comparisons, the sexual orientation variable was successfully coded into a dichotomous variable, where 54.3% (*n* = 50) of valid cases identified as sexual minorities and 45.7% (*n* = 42) as heterosexual. Racial identities of the population were analyzed, with 60.4% (*n* = 58) of participants identifying as POC and 39.6% (*n* = 38) identifying as white.

### 3.2. Bivariate Correlations

Bivariate Pearson correlations were examined among all primary continuous variables (see Table 2). Child age was positively associated with depressive symptoms (*r* = 0.26, *p* < 0.05) and negatively associated with social competence (*r* = −0.30, *p* < 0.01) and peer attachment (*r* = −0.23, *p* < 0.05).

Adaptive coping showed significant positive correlations with avoidant coping (*r* = 0.49, *p* < 0.01), anxiety (*r* = 0.28, *p* < 0.01), and social competence (*r* = 0.21, *p* < 0.05). Avoidant coping was significantly associated with higher anxiety (*r* = 0.57, *p* < 0.01), higher depressive symptoms (*r* = 0.68, *p* < 0.01), and lower peer attachment (*r* = −0.25, *p* < 0.05).

Anxiety demonstrated strong positive correlations with depression (*r* = 0.74, *p* < 0.01) and avoidant coping (*r* = 0.57, *p* < 0.01) and a negative correlation with peer attachment (*r* = −0.27, *p* < 0.01). Depression was negatively associated with social competence (*r* = −0.39, *p* < 0.01) and peer attachment (*r* = −0.53, *p* < 0.01).

Social competence was positively correlated with peer attachment (*r* = 0.37, *p* < 0.01). Peer attachment also demonstrated significant negative associations with avoidant coping (*r* = −0.25, *p* < 0.05), anxiety (*r* = −0.27, *p* < 0.01), and depressive symptoms (*r* = −0.53, *p* < 0.01).

See Table 2 for further information on bivariate correlations.

### 3.3. Linear Regressions

#### 3.3.1. Predicting Depression

A multiple linear regression was conducted to examine whether peer attachment predicted depressive symptoms after controlling for age and avoidant coping. The overall model was statistically significant (F(3, 87) = 40.70, *p* < 0.001), accounting for 58.4% of the variance in depression scores. Peer attachment emerged as a significant negative predictor (β = −0.351, *p* < 0.001), indicating that higher quality peer relationships were associated with lower depressive symptoms. Avoidant coping was a strong positive predictor (β = 0.570, *p* < 0.001), suggesting that greater use of avoidance strategies was linked to higher depression. Age was not a significant predictor in the model (β = 0.053, *p* = 0.458).

#### 3.3.2. Predicting Anxiety

A second regression analysis examined predictors of anxiety symptoms, using standardized scores for coping and peer attachment. The model was statistically significant (*F*(3, 87) = 15.38, *p* < 0.001), explaining a substantial 34.7% of the variance in anxiety. Standardized avoidant coping was a significant positive predictor (β = 0.530, *p* < 0.001). However, standardized peer attachment did not reach statistical significance as a predictor of anxiety (β = −0.161, *p* = 0.161). Age was not a significant predictor in this model.

#### 3.3.3. Predicting Adaptive Coping

A third regression tested whether peer attachment, age, and social competence predicted adaptive coping strategies. The model was not statistically significant (*F*(3, 84) = 1.71, *p* = 0.171), accounting for only 5.8% of the variance. None of the predictors—peer attachment, age, or social competence—significantly predicted adaptive coping.

### 3.4. Hierarchical Regression: Testing Moderation

A hierarchical regression was conducted to test whether peer attachment moderated the relationship between avoidant coping and depression. Step 1 included the main effects of standardized avoidant coping, standardized peer attachment, and age, which accounted for 58.4% of the variance. In Step 2, the interaction term between standardized avoidant coping and standardized peer attachment was added. The interaction term approached but did not reach statistical significance (β = 0.130, *p* = 0.061), with the model explaining an additional 1.7% of variance (Δ*R*^2^ = 0.017). To clarify the nature of the interaction, simple slopes were examined at low, medium, and high levels of peer attachment. The simple slope of avoidant coping predicting depressive symptoms was 3.78 at low peer attachment, 7.42 at medium peer attachment, and 6.38 at high peer attachment.

### 3.5. Covariate Testing

#### 3.5.1. Sexual Identity Comparisons

Independent-samples *t*-tests revealed no statistically significant differences between sexual minority (*n* = 50) and heterosexual (*n* = 42) adolescents on peer attachment, *t*(90) = 0.114, *p* = 0.909; anxiety symptoms, *t*(90) = −1.732, *p* = 0.087; depressive symptoms, *t*(87) = −1.916, *p* = 0.059; or adaptive coping, *t*(85) = −1.438, *p* = 0.154. Effect sizes for these comparisons were uniformly small. The largest observed effect was for depressive symptoms (Cohen’s *d* = −0.408), though this difference was not statistically significant.

#### 3.5.2. Gender Identity Comparisons

Similarly, no significant differences were found between cisgender (*n* = 80) and gender-expansive (*n* = 17) adolescents on peer attachment, *t*(95) = 0.713, *p* = 0.478; anxiety symptoms, *t*(95) = −0.391, *p* = 0.697; depressive symptoms, *t*(91) = −1.423, *p* = 0.158; or adaptive coping, *t*(91) = −0.255, *p* = 0.799. Corresponding effect sizes (Cohen’s d) for these comparisons ranged from −0.362 to 0.148, all within the small range.

#### 3.5.3. Racial Identity Comparisons

Analyses comparing youth of color (*n* = 60) and white youth (*n* = 37) also showed no significant differences on peer attachment, *t*(95) = −0.821, *p* = 0.414; anxiety symptoms, *t*(95) = 0.386, *p* = 0.700; depressive symptoms, *t*(91) = 0.108, *p* = 0.914; or adaptive coping, *t*(91) = −0.807, *p* = 0.422. The effect sizes for these comparisons were negligible to small, with Cohen’s d-values ranging from −0.168 to 0.089.

#### 3.5.4. Summary of Group Differences

Across all identity-based comparisons—sexual, gender, and racial—no statistically significant disparities emerged in peer attachment, mental health symptoms, or coping styles. All computed effect sizes (Cohen’s d, Hedges’ correction, and Glass’s delta) fell within the small range, indicating minimal practical differences between groups on the variables in this sample. Although no statistically significant differences emerged across identity groups, descriptive means indicated that sexual minority adolescents reported higher depressive symptom scores compared to heterosexual adolescents. Additionally, the mean depressive symptom score for the overall sample was above the established clinical cutoff.

## 4. Discussion

The current study investigated the role of friendships and peer relationships in the mental health and coping of adolescents with LGBTQ+ parents, a population whose extra-familial social world remains understudied. Overall, the findings suggest that higher quality peer relationships are linked with fewer depressive symptoms, and avoidant coping is connected to higher symptoms of anxiety. However, the results also present nuanced complexities, particularly regarding anxiety, adaptive coping, and the absence of within-group disparities, which warrant careful interpretation in light of existing literature and theoretical frameworks.

Descriptive patterns in the sample provide important context for interpreting the findings. On average, adolescents reported mild levels of anxiety, indicating generally low anxiety in this population. In contrast, mean depressive symptoms exceeded clinical cutoffs, suggesting elevated levels of depressive affect relative to general population norms. Adolescents also reported moderate levels of social competence and moderate-to-high levels of peer attachment, indicating that most youth perceived their friendships as warm, supportive, and trusting. These descriptive findings suggest that while adolescents with LGBTQ+ parents generally exhibit low anxiety and strong peer relationships, depressive symptoms may be a more prominent area of concern in this population. These patterns are consistent with prior research showing that adolescents with LGBTQ+ parents generally demonstrate strong social functioning and positive peer relationships (Gartrell & Bos, 2010). Though it is unclear why this sample of adolescents experienced high depression rates, it is possible that it could be attributed to general rising trends of depression among U.S. adolescents in recent years (Centers for Disease Control and Prevention, 2024), or perhaps it could be due to minority stress faced for being the children of LGBTQ+ parents in an increasingly hostile anti-LGBTQ+ climate (Goldberg & Smith, 2024). Although identity-based differences were not statistically significant, the descriptive pattern suggests that sexual minority adolescents may experience higher levels of depressive symptoms than their heterosexual peers, consistent with minority stress frameworks. Given that the overall sample mean exceeded clinical thresholds, and sexual minority youth reported even higher scores, these findings highlight a potentially meaningful trend that warrants further attention. Additionally, while the interaction between avoidant coping and peer attachment was only marginally significant, the simple slopes indicated that avoidant coping was associated with depressive symptoms across levels of peer attachment, with a somewhat stronger association at higher attachment levels. This pattern may point to nuanced relational dynamics that could inform future research.

Beyond the descriptive findings, the correlational patterns among the study variables offer deeper insight into the social and psychological experiences of adolescents with LGBTQ+ parents. Peer attachment demonstrated significant associations with multiple indicators of well-being. Higher-quality peer attachment was strongly associated with lower depressive symptoms and lower anxiety, as well as higher social competence, suggesting that supportive and trusting friendships are linked to more positive adjustment across emotional and social domains. This aligns with ecological and social support frameworks that emphasize peers as an increasingly central developmental context during adolescence (Bandura & Walters, 1977; Bronfenbrenner & Morris, 2007) and extend existing literature to a sample of adolescents from LGBTQ+-parent families. Avoidant coping showed strong associations in our correlation analyses, exhibiting strong links with both greater depression and anxiety. These findings further reinforce the substantial psychological risks associated with disengagement-based coping strategies (Compas et al., 2017), including among adolescents with LGBTQ+ parents. The moderate negative correlation between avoidant coping and peer attachment suggests that adolescents who rely more on avoidance may struggle to form or maintain close, supportive friendships or that youth with stronger peer bonds are less likely to use avoidant strategies (Armsden & Greenberg, 1987).

Adaptive coping, in contrast, showed positive associations with social competence and anxiety, as well as with avoidant coping. These patterns suggest that while adolescents may use a mix of coping responses, adaptive strategies alone may not fully buffer distress. That adaptive coping was positively correlated with social competence is consistent with developmental literature linking prosocial engagement to more flexible coping repertoires (Zimmer-Gembeck & Skinner, 2011). Depressive symptoms showed strong positive associations with anxiety and negative associations with social competence and peer attachment. Together, these results reinforce the interplay between internalizing symptoms and relational support. Adolescents who perceive themselves as more socially capable and more securely connected to peers appear less likely to experience elevated depressive symptoms, consistent with prior work showing that strong peer relationships predict better emotional adjustment and lower internalizing distress (Bukowski et al., 2011; Laursen & Collins, 2009). These findings also align with attachment-based models of peer relationships (Armsden & Greenberg, 1987) and with ecological perspectives emphasizing the protective function of supportive peer microsystems in adolescence (Bronfenbrenner & Morris, 2007). Moreover, the pattern echoes minority stress frameworks, which highlight social support as a key buffer against the psychological toll of stigmatization among youth in LGBTQ+ family contexts (Patterson, 2017).

Finally, demographic variables showed a small set of meaningful associations: age was positively correlated with depressive symptoms and negatively correlated with peer attachment and social competence. These patterns reflect broader developmental trends, wherein older adolescents often report increases in internalizing distress alongside shifts in peer closeness and social roles (Hamza & Willoughby, 2011; Patton, 2016).

The most robust finding of this study supports this hypothesis in that there was a strong, negative association between peer attachment and depressive symptoms. The regression model predicting depression accounted for a substantial portion of the variance (58.4%), with peer attachment emerging as a significant, unique predictor even after controlling for avoidant coping. For adolescents with LGBTQ+ parents, who may routinely navigate curiosity, questioning, or stigma related to their family structure (Goldberg & Smith, 2024), high-quality friendships appear to serve as a powerful, extra-familial buffer. This result aligns directly with the central hypothesis and provides strong empirical support for Minority Stress Theory (Brooks, 1981; Meyer, 1995), which posits social support as a critical buffer against the negative psychological impacts of stigma. This finding echoes prior research emphasizing the importance of social support for adolescent adjustment but specifically extends it to the context of peer relationships for this population (Sumontha et al., 2017).

The test of the buffering hypothesis that peer attachment would weaken the link between avoidant coping and depression yielded a marginal, non-significant interaction. While the effect was in the predicted direction, its borderline significance suggests a potential but not definitive protective role. A larger sample with greater statistical power might clarify this relationship. Nevertheless, the trend supports the ecological perspective that positive functioning emerges from complex interactions between risk and protective factors across different systems (Bronfenbrenner & Morris, 2007). The mesosystemic link between the peer microsystem (attachment) and the individual’s coping style appears important, warranting further exploration with more nuanced measures of peer interactions and coping modeling.

Across all identity-based comparisons, peer attachment levels appeared remarkably stable, suggesting that adolescents with LGBTQ+ parents experience similarly strong and supportive friendships regardless of their sexual identity, gender identity, or racial background. The lack of significant differences—and uniformly small effect sizes—indicates that the quality of adolescents’ peer relationships does not vary meaningfully across demographic groups (Prinstein & La Greca, 2004; Rubin et al., 2006). This consistency highlights peer attachment as a shared relational strength in this population, pointing to the possibility that adolescents with LGBTQ+ parents may cultivate resilient, trusting peer connections irrespective of their intersecting identities. In addition, broader developmental research underscores the importance of adolescents’ social identity competencies for positive adjustment across contexts. For example, Umaña-Taylor (2023) highlights that normative identity development processes, such as ethnic–racial identity exploration and cohesion, are linked with better psychological functioning and resilience during adolescence. Although focused on ethnic–racial identity, this work aligns with the present findings by illustrating that developmental competencies linked to peers and social contexts support adjustment across diverse youth, reinforcing the idea that supportive peer attachments are widely accessible developmental assets.

### Strengths, Limitations, Future Research Directions, and Implications

Several limitations should be considered. First, the cross-sectional, correlational design prevents causal inferences. For example, we cannot determine whether strong peer attachment leads to lower depression or whether adolescents with lower depression are more capable of forming strong attachments. Longitudinal research is needed to untangle these temporal relationships. Additionally, the sample, while diverse, was one of convenience and modest size, potentially limiting generalizability and statistical power, particularly for subgroup analyses and detecting interaction effects. The marginal moderation effect is a key example. Further, reliance on self-report measures introduces the potential for some common-method bias. Future studies would benefit from multi-informant reports (e.g., parent or teacher reports) and observational measures of peer interaction quality. Another important point is that, although we examined group differences across sexual, gender, and racial identities using separate *t*-tests (see Section 3.5.1, Section 3.5.2 and Section 3.5.3), we did not include minoritized identities as covariates or moderators in our primary regression models. We agree that, ideally, we would have tested whether adolescents’ sexual minority status, gender-expansive identity, or race moderated the relationship between peer attachment and mental health. However, given our limited statistical power (N = 98) and the modest cell sizes within these subgroups (e.g., *n* = 17 for gender-expansive youth), we were unable to include these interaction terms without risking model instability and overfitting. Consequently, our null findings from the subgroup *t*-tests should not be interpreted as evidence that peer attachment functions identically across these groups. Rather, these findings indicate only that we did not detect differences in this specific, underpowered sample. We therefore caution against concluding that peer attachment is ‘remarkably stable’ across minoritized subgroups. Future research with larger, more diverse samples is needed to rigorously test whether adolescents’ own LGBTQ+ or racial/ethnic minoritized status moderates the protective associations documented here. One final limitation was the reliability statistics of the social competence subscale. Our team ran an item analysis on this scale and found that one question (i.e., “How well do you do things alone?”) did not load well with other items. Once this item was deleted and replaced with the average of the other 5 items, analyses were re-run. This, however, did not change the strength or direction of relationships, so we decided to retain the original scale.

Taken together, the present findings also highlight several strengths and contributions that advance understanding of adolescents with LGBTQ+ parents. A major strength lies in our study’s explicit emphasis on peer processes, with our findings implying the significant connection peer relationships have with negative mental health symptoms, such as depression. The robust association between peer attachment and depressive symptoms illustrates how everyday relational experiences of support from friends may have the potential to serve as accessible buffers against internalizing distress for youth who may navigate questions or stigma related to their family structure. Importantly, the consistency of peer attachment across sexual, gender, and racial subgroups suggests that high-quality friendships represent a relational strength that transcends demographic variation, offering a promising leverage point for prevention and intervention. These implications extend to practice: schools, community programs, and mental health professionals can play a crucial role in cultivating supportive peer environments and LGBTQ+-affirming climates. By foregrounding both risk and relational resilience, this study contributes to a strengths-based understanding of adolescents with LGBTQ+ parents and highlights peer relationships as a developmentally grounded, broadly accessible target for supporting well-being.

Future research should build on these findings by investigating the specific behaviors and content of supportive peer interactions. What do friends actually say or do that buffers distress? Qualitative methodologies could richly illuminate these processes. Furthermore, research should continue to explore potential differences for “second-generation” queer youth, perhaps using larger samples or more specific measures of LGBTQ+-specific peer support and identity affirmation (Goldberg & Smith, 2024; Kuvalanka & Goldberg, 2009). Finally, it would be worth examining the interplay between family and peer systems, the mesosystem. How do parenting practices in LGBTQ+ parent families relate to an adolescent’s peer network, and how do experiences with peers get communicated and processed within the family?

## 5. Conclusions

This study makes a meaningful contribution by shifting the focus beyond the family walls to the peer relationships of adolescents with LGBTQ+ parents. Because adolescence is a developmental period marked by increasing autonomy, heightened sensitivity to social evaluation, and a growing reliance on friendships for emotional support and identity exploration, peers play a uniquely influential role in youths’ adjustment. Within this context and in line with ecological, attachment, and minority stress frameworks, the present findings underscore that high-quality peer attachments have the potential to function as a critical protective asset, particularly for adolescents who may navigate curiosity or stigma related to their family structure, with strong friendships showing a strong association with lower depressive symptoms. This affirms the theoretical importance of the peer microsystem as a developmental context for mental health-related resilience. Simultaneously, the findings highlight the universal and powerful risk posed by avoidant coping strategies. Practically, this suggests that interventions aimed at supporting the mental health of adolescents in LGBTQ+-parented families should have a dual focus: fostering positive peer relationship skills and reducing reliance on avoidant coping, perhaps through developmentally appropriate psychoeducation and coping skills training. Importantly, the lack of within-group disparities based on personal identities offers a positive narrative about the capacity for healthy adjustment among these youth. Ultimately, by recognizing and strengthening the protective web of friendships, we can better support the well-being of adolescents navigating the unique and rewarding landscape of growing up in an LGBTQ+-parented family.

## Figures and Tables

**Table 1:** Sample Demographics.

Demographics	
Characteristic	Count (Percentage)
Race	
white	38 (38.7)
Hispanic/Latino	18 (18.3)
Asian/Pacific Islander	13 (13.2)
Black	8 (8.2)
Multiracial	11 (11.2)
African American/Black	4 (4.1)
Middle Eastern	3 (3.1)
Native American	1 (1.0)
Missing	2 (2.0)
Gender Identity	
CM (Cis Men)	51 (52.0)
CF (Cis Women)	29 (29.6)
NB (Nonbinary)	7 (7.1)
GNC (Gender Nonconforming)	3 (3.1)
TM (Transmasculine)	3 (3.1)
TW (Trans Woman)	2 (2.0)
Gender-Fluid	1 (1.0)
Missing	2 (2.0)
Sexual Orientation	
Heterosexual	42 (42.9)
Bisexual	24 (24.5)
Gay	8 (8.2)
Pansexual	7 (7.1)
Lesbian	4 (4.1)
Queer	4 (4.1)
Asexual	3 (3.1)
Missing	6 (6.1)

*Note*. Total *N* = 98.

**Table 2:** Descriptive Information for and correlations among variables of interest and age.

Variable	*n*	*M*	*SD*	1	2	3	4	5	6
1. Child Age	96	18.18	1.52	-	-	-	-	-	-
2. Adaptive Coping	93	2.35	0.57	−0.18	-	-	-	-	-
3. Avoidant Coping	93	1.99	0.49	0.14	0.49 **	-	-	-	-
4. Anxiety	97	7.04	5.64	0.17	0.28 **	0.57 **	-	-	-
5. Depression	93	20.26	11.77	0.26 *	0.08	0.68 **	0.74 **	-	-
6. Social Competence	95	41.57	12.03	−0.30 **	0.21 *	−0.17	−0.10	−0.39 **	-
7. Peer Attachment	97	91.26	19.95	−0.23 *	0.11	−0.25 *	−0.27 **	−0.53 **	0.37 **

*Note*. * *p* < 0.05. ** *p* < 0.01.

## Data Availability

Although the data presented in this study are not publicly available due to privacy and ethical restrictions, they are available upon reasonable request from the authors (R.H.F. is PI).
